# miR-129 as a Molecular Biomarker in Gastric Cancer and Its Association with Neurodegenerative and Vascular Pathology

**DOI:** 10.3390/life15101603

**Published:** 2025-10-14

**Authors:** Sabrina Birsan, Adrian-Gheorghe Boicean, Paula Anderco, Cristian Ichim, Samuel Bogdan Todor, Roman Filip Iulian, Blanca Grama, Anca-Rafila Stîngaciu, Olga Brusnic, Tiberia Ilias, Corina Roman-Filip

**Affiliations:** 1 Faculty of Medicine, University Lucian Blaga, 550024 Sibiu, Romania; sabrinaandreea.marinca@ulbsibiu.ro (S.B.); paula.anderco@ulbsibiu.ro (P.A.); cristian.ichim@ulbsibiu.ro (C.I.); samuelbogdant@gmail.com (S.B.T.); stingaciu.anca.r@gmail.com (A.-R.S.); corina.roman@ulbsibiu.ro (C.R.-F.); 2 Clinical Department, Faculty of Medicine, University of Medicine ‘George Emil Palade’, 540139 Targu Mures, Romania; iulian_roman2009@yahoo.com (R.F.I.); brusnic_olga@yahoo.com (O.B.); 3 Faculty of Psychology, University Lucian Blaga of Sibiu, 550024 Sibiu, Romania; blanca.grama@ulbsibiu.ro; 4 Department of Gastroenterology, Faculty of Medicine, University of Oradea, 410087 Oradea, Romania; tiberia_ilias@uoradea.ro

**Keywords:** gastric cancer, biomarkers, miRNA, screening, neuroinflammation

## Abstract

Background: MicroRNA-129 (miR-129) is a tumor suppressor involved in regulating oncogenic pathways, but its role in gastric adenocarcinoma and its potential connections to vascular and neurological dysfunction remain insufficiently defined. Objectives: To assess gastric juice-derived miR-129 as a diagnostic and prognostic biomarker for gastric cancer and to explore its associations with systemic inflammation, vascular impairment, and neurodegenerative changes. Methods: A prospective study was conducted in 38 patients undergoing upper gastrointestinal endoscopy (22 with histologically confirmed gastric adenocarcinoma, 16 controls). Gastric juice was aspirated prior to biopsy, and miR-129-2-3p expression was quantified by means of RT-qPCR normalized to U6 RNA. Tumor stage, serum biomarkers (CEA, CA 19-9, LDH, and CRP), carotid index (Doppler ultrasound), and neuroimaging (MRI) were recorded. Statistical analyses included ANOVA, Mann–Whitney U, ROC curve analysis, and correlation testing. Results: miR-129 expression was significantly reduced in gastric cancer compared with controls (ANOVA: F(3,34) = 3.70, *p* = 0.021, η^2^ = 0.25). ΔCt values increased progressively from controls to T2–T4 tumors, indicating stage-dependent downregulation. ROC analysis demonstrated moderate diagnostic performance (AUC = 0.75, 95% CI 0.54–0.92). Lower miR-129 levels correlated inversely with serum tumor markers (CEA, CA 19-9), LDH, and CRP. Patients with elevated carotid index (>1.3) and abnormal brain imaging findings exhibited significantly lower miR-129 expression (both *p* < 0.05). Conclusion: Gastric juice-derived miR-129 is downregulated in gastric adenocarcinoma, with progressive decline across tumor stages. Its inverse association with systemic tumor and inflammatory markers, as well as vascular and neurological impairment, suggests that miR-129 may function as a minimally invasive, multi-system biomarker for integrated cancer and vascular–neurological risk assessment.

## 1. Introduction

Gastric cancer remains one of the leading causes of cancer-related morbidity and mortality worldwide, with a particularly high burden in Eastern Europe and East Asia [1,2,3,4,5]. Despite advances in diagnostic endoscopy and imaging techniques, most patients are diagnosed at an advanced stage, when curative treatment options are limited and prognosis is poor. The identification of reliable, minimally invasive biomarkers for early detection, staging, and prognosis is therefore a pressing clinical need [1,2,3,4,5].

MicroRNAs (miRNAs) are small non-coding RNA molecules that regulate gene expression at the post-transcriptional level and play key roles in diverse biological processes, including cell proliferation, apoptosis, differentiation, and migration. Aberrant expression of specific miRNAs has been documented in various malignancies, including gastric cancer, where they can act as either oncogenes or tumor suppressors. Because of their stability in body fluids and disease-specific expression patterns, miRNAs have emerged as promising diagnostic and prognostic tools [6,7,8,9,10,11,12,13,14,15,16,17,18,19,20,21,22,23,24,25].

Among them, miR-129 (hsa-miR-129-2-3p) has been identified as a tumor suppressor implicated in the regulation of oncogenic pathways, including HMGB1, BCL2, and thymidylate synthase. Previous studies have reported its downregulation in gastric and colorectal cancers, correlating with poor differentiation, advanced stage, and reduced survival. However, most research to date has focused on tissue or plasma samples, whereas the potential utility of gastric juice—a biologically relevant and minimally invasive matrix—for miR-129 analysis has been largely unexplored [25,26,27,28,29,30,31,32,33,34].

Beyond its role in tumorigenesis, accumulating evidence suggests that miRNAs may also participate in systemic processes such as vascular dysfunction and neurodegeneration, which share pathogenic mechanisms with cancer, including chronic inflammation, oxidative stress, and endothelial injury. This opens the possibility that a single miRNA biomarker could serve as a molecular link between oncology, cardiovascular disease, and neurological disorders [34,35,36,37,38,39,40,41,42,43,44,45,46,47,48,49,50].

In this study, we investigated the expression of miR-129 in gastric juice from patients with gastric adenocarcinoma and compared it with non-cancer controls. We further examined its associations with tumor stage, established tumor markers (CEA and CA 19-9), vascular impairment (carotid index), and brain imaging abnormalities. Our aim was to evaluate miR-129 not only as a biomarker for gastric cancer diagnosis and prognosis but also as a potential indicator of systemic vascular and neurological pathology.

## 2. Materials and Methods

This prospective, observational study was conducted at the Sibiu Clinical Emergency Hospital, Gastroenterology Department, in collaboration with the Molecular Biology Laboratory of the Applied Ecology Research Center, Lucian Blaga University of Sibiu, Romania. The protocol followed the ethical principles of the Declaration of Helsinki and was approved by the Institutional Ethics Committee. Written informed consent was obtained from all participants.

A total of 38 patients undergoing upper gastrointestinal endoscopy were enrolled: 22 with histologically confirmed gastric adenocarcinoma (study group) and 16 controls (with minimal superficial gastritis treated as non-malignant controls).

Inclusion criteria: age ≥ 18 years, histopathological confirmation of gastric adenocarcinoma (study group) or healthy patients (controls), endoscopic description of the lesion, availability of MRI scans, and carotid Doppler evaluation. Exclusion criteria: other active malignancies, prior chemotherapy for gastric cancer, brain metastases or primary brain tumors, and autoimmune neurological disorders.

Data collected included demographics, comorbidities (diabetes, neurological disease, and cardiovascular disease), clinical manifestations (anemia, weight loss, early satiety, and epigastric pain), and laboratory parameters (hemoglobin, CEA, CA 19-9, LDH, and CRP). Vascular status was assessed by carotid Doppler ultrasound (carotid index), while neurodegenerative changes were evaluated by MRI, and cognitive status by the Mini-Mental State Examination (MMSE). Tumor depth and TNM staging were determined by endoscopic ultrasound.

### 2.1. MiRNA Extraction and Quantification

Gastric juice was aspirated endoscopically prior to biopsy. Total RNA was extracted from 500 μL of gastric juice using TRIzol reagent (Invitrogen/Thermo Fisher Scientific, Carlsbad, CA, USA) and eluted in 40 μL ultrapure water. RNA yield and purity were assessed using the Qubit microRNA assay. Reverse transcription was performed with the TaqMan MicroRNA Reverse Transcription Kit Thermo Fisher Scientific (under the Applied Biosystems™ brand), Carlsbad, CA, USA, using primers specific for hsa-miR-129-2-3p. qPCR was carried out with TaqMan Universal Master Mix II (Thermo Fisher Scientific (under the Applied Biosystems™ brand), Carlsbad, CA, USA), no UNG, on a standard thermocycling protocol (95 °C for 10 min, followed by 40 cycles of 95 °C/15 s and 60 °C/1 min). U6 RNA served as an internal reference. Ct values were used to calculate relative expression (ΔCt = Ct_miR-129 − Ct_U6).

### 2.2. Statistical Analysis

All analyses were performed using R (v4.2.1) and SPSS (v27). Continuous variables were expressed as the mean ± standard deviation (SD) or median with interquartile range (IQR), while categorical variables were summarized as counts and percentages. Normality of ΔCt values was assessed with the Shapiro–Wilk test, and homogeneity of variances was tested using Levene’s test. Depending on distributional assumptions, comparisons between two groups were performed with the independent-samples *t*-test or the Mann–Whitney U test (with rank-biserial correlation r reported as effect size). For comparisons across multiple groups, one-way ANOVA with Tukey’s HSD post-hoc was used when assumptions were met; otherwise, the Kruskal–Wallis test with Bonferroni-adjusted pairwise Mann–Whitney tests was applied. Global effect sizes were reported as η^2^ (eta squared) for ANOVA and ε^2^ (epsilon squared) for Kruskal–Wallis. Diagnostic accuracy was assessed using ROC curve analysis with calculation of the area under the curve (AUC) and 95% confidence intervals (bootstrap method). The optimal cutoff was determined using the Youden index, and diagnostic performance was summarized with sensitivity, specificity, positive predictive value (PPV), and negative predictive value (NPV), including 95% CIs. Associations between miR-129 expression and continuous biomarkers (CEA, CA 19-9, LDH, CRP) (Table 1) were assessed with Pearson’s correlation for normally distributed variables and Spearman’s rho otherwise. Associations with categorical variables (e.g., neuroimaging abnormalities, carotid index categories) were tested with the chi-square test or Fisher’s exact test when expected cell counts were <5. A two-tailed *p* < 0.05 was considered statistically significant. 

## 3. Results

### 3.1. General Characteristics of Individuals

In this prospective study, consisting of 22 patients with gastric adenocarcinoma and 16 control subjects, significant differences were observed between the two groups across multiple clinical and biochemical parameters (Table 1). Patients with gastric adenocarcinoma were older compared to the control group (median age 67.5 vs. 55 years, *p* = 0.001) and demonstrated lower cognitive performance, reflected by significantly reduced MMSE scores (median 19 vs. 24.5, *p* = 0.003). No statistically significant differences were found regarding gender distribution (*p* = 0.132) or *residency (urban/rural) (*p* = 0.556). However, a marked difference was observed in carotid intima-media thickness (cIMT), with higher values (>1.3 mm) more prevalent among adenocarcinoma patients (*p* = 0.005). Similarly, abnormal MRI findings were significantly more frequent in the gastric adenocarcinoma group (*p* = 0.003). Regarding biochemical markers, patients with gastric adenocarcinoma presented significantly elevated levels of total lipids, triglycerides, LDL, CEA, CA19.9, PCR, and LDH (all *p* < 0.01), as well as decreased Ht (Hematocrit), MCHC (Mean Corpuscular Hemoglobin Concentration), MCV (average volume (size) of a red blood cell), and Hb (Hemoglobin) values (all *p* < 0.001). HDL levels did not differ significantly between groups (*p* = 0.737). Additionally, the log2-scaled Fold variable showed a significant decrease in the adenocarcinoma group (median 3.15 vs. 4.94, *p* = 0.044) (Table 1).

**Table 1:** Demographic and clinical characteristics of the patients.

General Characteristics of the Patients				
Variable		Adenocarcinoma	Control	*p*-Value
Age (years)		67.5 (61–73)	55 (52–59)	0.001
MMSE		19 (10–20)	24.5 (20–25.5)	0.003
Gender	Male	15 (68.2%)	7 (43.8%)	0.132
	Female	7 (31.8%)	9 (56.2%)	
Residency	Urban	17 (77.3%)	11 (68.8%)	0.556
	Rural	5 (22.7%)	5 (31.2%)	
cIMT	0.9–1.3 mm	5 (22.7%)	11 (68.8%)	0.005
	>1.3 mm	17 (77.3%)	5 (31.2%)	
MRI	Negative	0 (0.0%)	6 (37.5%)	0.003
	Positive	22 (100.0%)	10 (62.5%)	
Total Lipids (mg/dL)		900 (840–1000)	550 (400–825)	<0.001
Triglycerides (mg/dL)		350 (200–500)	160 (140–260)	<0.001
HDL (mg/dL)		44 (40–50)	43 (42–48.5)	0.737
LDL (mg/dL)		178.5 (167–190)	149 (134.5–177)	0.002
Log_10_-Scaled		3.15 (2.15–4.18)	4.94 (3.12–5.35)	0.044
CEA (ng/mL)		15 (6–34)	4 (3–4.5)	<0.001
CA19-9 (U/mL)		50 (32–86)	9 (5–19.5)	<0.001
Hematocrit (%)		33.5 (29–37)	44.5 (42.5–45)	<0.001
MHC (g/dL)		29 (27–31)	35 (34–35.5)	<0.001
MCV (fl)		75 (68–78)	87.5 (85.5–90)	<0.001
Hemoglobin (g/dL)		8.25 (7–9.3)	13.4 (13–14)	<0.001
C-reactive protein (mg/L)		23.5 (17–32)	2.5 (2–3)	<0.001
LDH (U/L)		270 (240–300)	152.5 (139–168)	<0.001
ALP (U/L)		162 (88–185)	131.5 (121.5–142.5)	0.112

**Figure 1:**
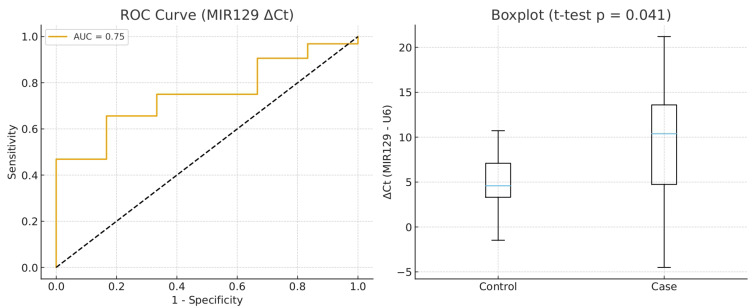
Diagnostic Performance and Expression Levels of miR-129 (ΔCt) in Gastric Adenocarcinoma.Left panel: ROC (Receiver Operating Characteristic) curve for miR-129 ΔCt values (miR-129 – U6), assessing the ability to distinguish between control and case groups. The Area Under the Curve (AUC) is 0.75, indicating moderate diagnostic accuracy. Right panel: Boxplot comparing miR-129 ΔCt values between control and case groups. A significant difference was observed using an unpaired *t*-test (*p* = 0.041), suggesting reduced miR-129 expression in the case group (higher ΔCt = lower expression).

These results suggest that miR129 ΔCt is significantly higher in gastric cancer patients than in controls, with moderate discriminatory accuracy (AUC = 0.75). Although sensitivity was moderate, specificity and PPV were high, indicating that elevated ΔCt strongly predicts disease presence in this sample. The diagnostic utility of MIR129 ΔCt (normalized to U6) was assessed in patients with gastric adenocarcinoma and controls. ROC curve analysis yielded an AUC of 0.75 (95% CI: 0.54–0.92), consistent with moderate discriminatory accuracy. The optimal threshold, determined by the Youden index, provided a sensitivity of 65.6% (95% CI: 48.3–79.6) and a specificity of 83.3% (95% CI: 43.6–97.0), with a PPV of 95.5% (95% CI: 78.2–99.2) and an NPV of 31.3% (95% CI: 14.2–55.6). Comparison of ΔCt values between groups showed higher levels in cancer patients (mean = 9.62) compared with controls (mean = 4.87). An independent samples *t*-test confirmed that this difference was statistically significant (t = 2.37, *p* = 0.041) (Figure 1) (Table 2).

**Figure 2:**
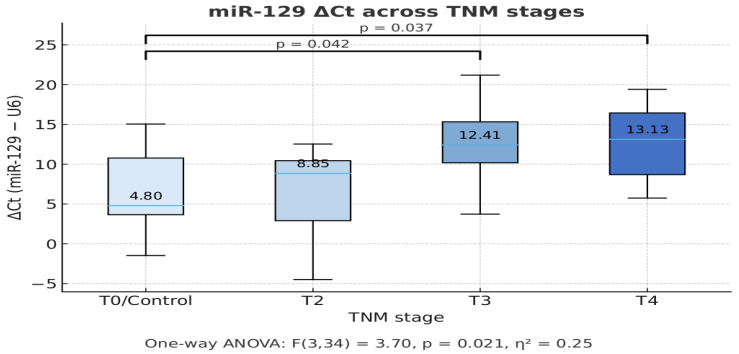
MiR-129 Expression Across TNM Stages in Gastric Adenocarcinoma.Boxplot showing the relative expression levels of miR-129 across different TNM stages of tumor progression, based on ΔCt values (miR-129–U6). Lower ΔCt values correspond to higher expression of miR-129. The expression of miR-129 was compared between control (T0) and cancer groups (T2, T3, T4). A one-way ANOVA revealed a significant overall difference in miR-129 expression across the stages (F(3,34) = 3.70, *p* = 0.021, η2 = 0.25). Post-hoc comparisons showed statistically significant differences between T0 vs. T3 (*p* = 0.042) and T0 vs. T4 (*p* = 0.037), indicating increased ΔCt (i.e., lower miR-129 expression) in advanced tumor stages.TNM stage: Tumor–Node–Metastasis classification system used for cancer staging: T0/Control: Non-tumoral or healthy tissue. T2, T3, T4: Progressive tumor stages (increasing size/invasion).

We investigated whether miR 129 ΔCt values differed across TNM stages of gastric cancer. Boxplot analysis (Figure 2) demonstrated increasing ΔCt values in higher tumor stages. Nonparametric testing using the Kruskal–Wallis test showed a significant difference between groups (H = 8.46, *p* = 0.037), and parametric testing confirmed this with a one-way ANOVA (F = 3.70, *p* = 0.021). Together, these results indicate that miR 129 expression (normalized to U6) increases with tumor stage, with higher ΔCt values observed in T3–T4 compared to controls and T2 tumors. (Figure 2.)

**Figure 3:**
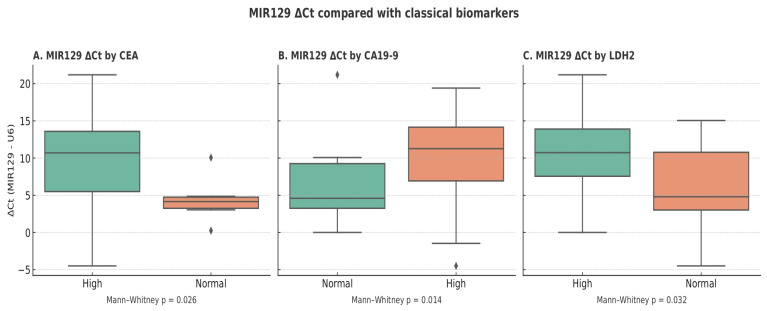
Association of miR-129 Expression (ΔCt) with Classical Tumor Biomarkers.This figure compares MIR129 expression levels (ΔCt relative to U6) with classical serum biomarkers—CEA, CA19-9, and LDH2. Each boxplot represents MIR129 ΔCt values in patients categorized according to whether the corresponding biomarker levels were high or within the normal range. Lower ΔCt values indicate higher MIR129 expression. Across all comparisons, MIR129 expression showed significant differences between the high and normal biomarker groups, as determined by the Mann–Whitney U test (*p* = 0.026 for CEA, *p* = 0.014 for CA19-9, and *p* = 0.032 for LDH2). These findings suggest that MIR129 expression may be associated with established tumor biomarkers, supporting its potential relevance in disease monitoring or diagnosis. (MIR129—MicroRNA-129, a small non-coding RNA involved in gene regulation. ΔCt—Delta Cycle Threshold; the difference between the Ct value of a target gene and a reference gene (used to normalize expression) U6—Small nuclear RNA U6, commonly used as an internal control for normalizing microRNA expression levels. CEA—Carcinoembryonic Antigen, a classical tumor biomarker often elevated in colorectal and other cancers. CA19-9—Carbohydrate Antigen 19-9, a serum biomarker associated primarily with pancreatic and gastrointestinal cancers, LDH2—Lactate Dehydrogenase Isoenzyme, an enzyme variant linked to tissue damage and certain malignancies).

We evaluated the relationship between miR129 ΔCt (normalized to U6) and established serum tumor markers. Patients with elevated CEA had significantly higher miR129 ΔCt values compared with those with normal CEA (mean 9.71 vs. 4.41; Mann–Whitney U = 151.0, *p* = 0.026). Similarly, patients with elevated CA19-9 also displayed higher miR129 ΔCt values compared with the normal group (mean 10.27 vs. 6.49; Mann–Whitney U = 250.0, *p* = 0.014) (Figure 3). These findings suggest that miR-129 expression is associated with classical tumor marker positivity in gastric cancer. Analysis of LDH2 status also revealed a significant difference, with MIR129 ΔCt elevated in LDH2-high compared to LDH2-normal patients (median 10.7 vs. 4.8, *p* = 0.032) (Figure 3).

**Figure 4:**
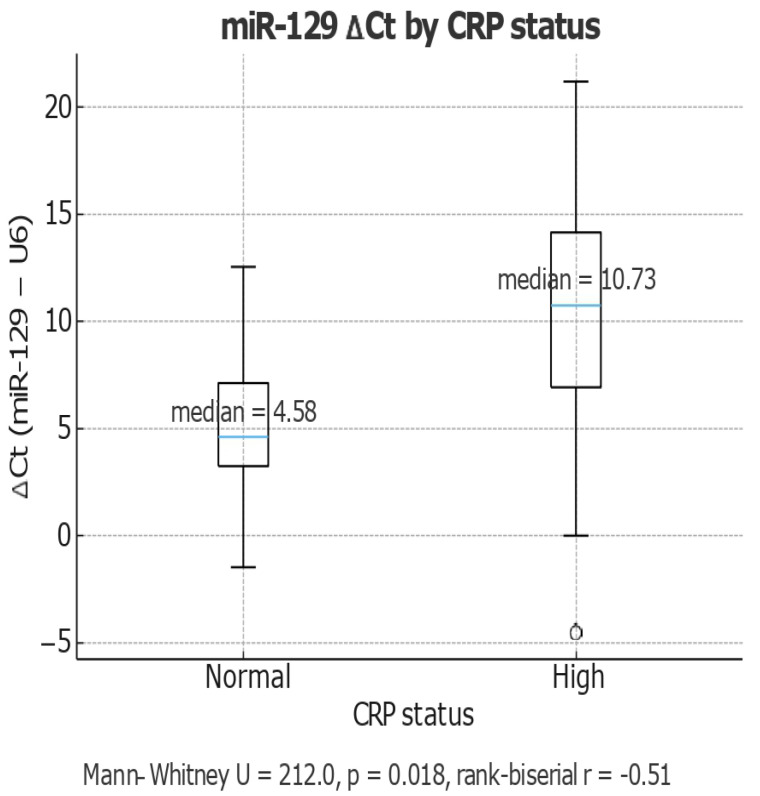
miR-129 Expression (ΔCt) in Relation to CRP Status. Boxplot comparing miR-129 expression levels (ΔCt) between subjects with normal and high C-reactive protein (CRP) status. The ΔCt values (miR-129 – U6) are significantly higher in the high CRP group (median = 10.73) compared to the normal CRP group (median = 4.58), indicating lower miR-129 expression in individuals with elevated inflammation markers (higher ΔCt = lower expression).

Boxplot analysis revealed that patients with high CRP levels had significantly increased miR-129 ΔCt values (median = 10.73) compared to those with normal CRP (median = 4.58), indicating an association between elevated systemic inflammation (Figure 4), also the significant associations with CA19-9, LDH, and CRP suggest that miR-129 could serve as a bridging biomarker between metabolic inflammation and cancer progression.

### 3.2. MIR-129 Impact on Neurodegenerative Diseases

Given the observed downregulation of miR-129 in gastric adenocarcinoma and its inverse correlation with systemic inflammatory and metabolic markers (CEA, CA 19-9, LDH, and CRP), we further explored whether these alterations extend beyond tumor biology to affect vascular and neurological function. Because chronic inflammation and oxidative stress represent shared molecular pathways linking cancer progression with neurodegeneration, miR-129 expression was analyzed in relation to cerebrovascular and cognitive parameters. To evaluate whether miR-129 expression differed according to brain imaging abnormalities or vascular impairment, patients were stratified based on MRI findings (normal vs. abnormal) and carotid index (≤1.3 vs. >1.3). Cognitive performance was assessed using the Mini-Mental State Examination (MMSE) to screen for cognitive impairment and monitor neurocognitive decline. Independent-samples *t*-tests (assuming equal variances) were applied to compare ΔCt values between groups, with results summarized in Table 3. Patients with abnormal MRI findings or an elevated carotid index (>1.3) exhibited significantly higher ΔCt values (*p* < 0.05), consistent with lower miR-129 expression. These findings suggest that miR-129 downregulation may not only reflect tumor burden but also indicate systemic processes linked to vascular dysfunction and neurodegenerative changes (Table 4).

The *t*-test applied to compare the miR-129 variable based on the mean carotid index revealed a statistically significant difference between the two analyzed groups (t = 8.60, *p* < 0.05). The group with an elevated carotid index (above 1.3) showed significantly higher ΔCt values, indicating reduced miR-129 expression in the context of vascular impairment.

Therefore, miR-129 is downregulated in patients with significant carotid artery involvement, suggesting its participation in molecular mechanisms related to vascular dysfunction and lipid accumulation.

**Figure 5:**
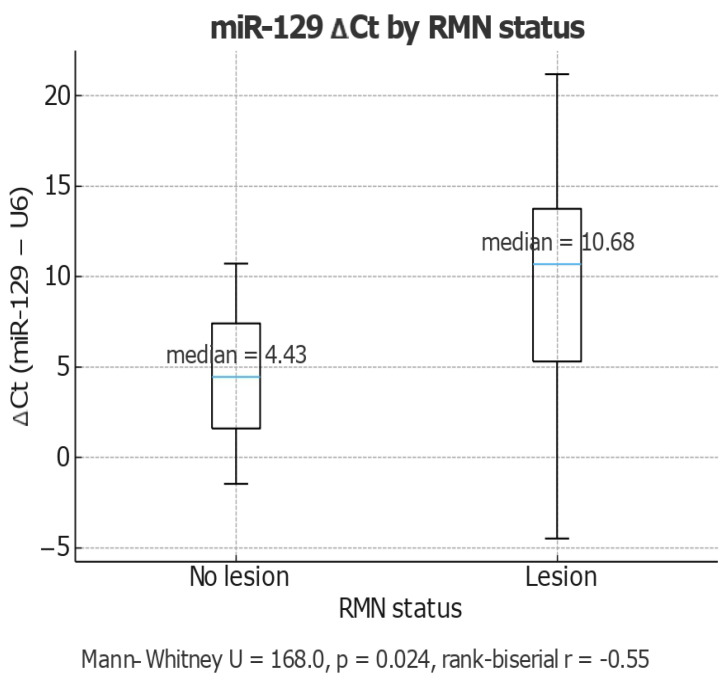
Boxplot MIR129/MRI Boxplot showing ΔCt values (miR-129-U6) in samples with and without lesions on MRI(magnetic resonance imaging; RMN in source data). Median expression was significantly lower in the no lesion group (median = 4.43) compared to the lesion group (median = 10.68) (Mann–Whitney U = 168.0, p = 0.024, rank-biserial r = −0.55).

The *t*-test applied to compare miR-129 expression based on brain imaging status (MRI) showed a significant difference between patients with normal MRI scans and those with pathological changes (t = 8.34, *p* < 0.05). The differences are also visible in Figure 5. The mean ΔCt value was significantly higher in patients with brain lesions, indicating lower miR-129 expression in this group. This result supports the idea that MIR-129 is downregulated in patients with imaging-visible brain damage, reflecting a functional role of this microRNA in neurovascular or neurodegenerative mechanisms.

Analysis of miR-129 ΔCt values stratified by MRI status revealed a significant difference between patients with and without brain lesions. The median ΔCt was 4.43 in the no-lesion group and 10.68 in the lesion group, reflecting markedly lower miR-129 expression in patients with neuroimaging abnormalities. A Mann–Whitney test confirmed the statistical significance of this difference (U = 168.0, *p* = 0.024), with a moderate-to-strong negative effect size (rank-biserial r = −0.55). These findings suggest that reduced miR-129 expression is strongly associated with the presence of neurodegenerative lesions on MRI. The area under the curve (AUC) was 0.74 (95% CI: 0.56–0.87), the optimal probability cutoff (Youden index = 0.38) yielded a sensitivity of 70.0% and specificity of 78.6%, supporting the potential of gastric juice-derived miR-129 as a noninvasive biomarker reflecting neuroimaging changes related to neurodegenerative processes or central nervous system injury in patients with gastric adenocarcinoma. The area under the ROC curve (AUC) with 95% confidence interval (CI) was estimated to quantify diagnostic accuracy. The statistical significance of the AUC was assessed against the null hypothesis of 0.5 (no discrimination). The optimal cut-off point was determined using the Youden Index (sensitivity + specificity − 1). Sensitivity and specificity were reported at this threshold. Our analysis demonstrated that higher relative miR-129 levels were associated with higher MMSE scores; the correlation was strong (Pearson r = 0.71, *p* < 0.0001), indicating that approximately 50% of the variance in MMSE performance could be explained by differences in miR-129 expression. ROC curve analysis was conducted to evaluate the diagnostic performance of normalized miR-129 expression for discriminating between participants with normal cognition (MMSE ≥ 24) and those with cognitive impairment (MMSE < 24). Relative expression levels of miR-129 were calculated using the 2^-ΔCT method (normalized to U6) (Figure 5).

## 4. Discussion

This study offers new insights into the role of miR-129 (hsa-miR-129-2-3p) as a potential molecular biomarker in gastric cancer, with additional implications in vascular impairment and neurodegenerative diseases. While the tumor-suppressive functions of miR-129 have been documented in various malignancies, particularly within the gastrointestinal tract, this research is among the first to evaluate its expression in gastric juice and explore its correlations with both tumor biology and neurovascular imaging parameters. Previous studies have investigated a range of gastric juice microRNAs (miRNAs) as diagnostic biomarkers. For instance, Yu et al. [12] identified miR-129 as a potential screening tool for gastric cancer, while other studies have emphasized miR-21, miR-106a, and miR-421 as biomarkers associated with tumor progression and therapy resistance. Additionally, Virgilio et al. [33] provided a systematic review underscoring both the promise and heterogeneity of miRNAs in gastric juice for cancer detection. Compared with these prior reports, our study contributes uniquely by extending the analysis of miR-129 to vascular and neuroimaging parameters, situating gastric juice miRNA profiling within a broader systemic disease framework. This multidimensional perspective positions miR-129 as a cross-disciplinary biomarker. Our findings show a significant downregulation of miR-129 in patients with gastric adenocarcinoma, relative to healthy controls. This result aligns with prior literature on miR-129’s role as a tumor suppressor, where it modulates targets such as HMGB1, BCL2, and thymidylate synthase, which are critical in cell proliferation, apoptosis, and DNA synthesis.

We also observed a progressive decline in miR-129 expression with increasing tumor depth (T2–T4), suggesting its utility in tumor staging and risk stratification. This trend was further validated by a significant inverse correlation with serum tumor markers—CEA and CA 19-9—highlighting miR-129’s diagnostic and prognostic potential. In addition to its oncologic relevance, our study explored vascular associations. A significant decrease in miR-129 levels was observed in patients with elevated carotid index values, indicating a potential link to vascular dysfunction. These findings suggest that miR-129 may play a role in endothelial integrity and atherosclerotic processes, possibly through inflammatory and oxidative stress pathways. Strikingly, patients with abnormal brain MRI findings—including lacunar infarcts and cerebral atrophy—also demonstrated reduced miR-129 expression. This is consistent with evidence from Kaurani et al. (2025) [46] and Loffreda et al. (2020) [47], who reported decreased miR-129-5p expression in neurodegenerative conditions such as frontotemporal dementia and ALS. Furthermore, our study found a positive correlation between miR-129 and cognitive performance, as measured by the MMSE, echoing the work of Qin et al. (2025) [48], who demonstrated that miR-129 enhances cognition by modulating astrocytic energy metabolism. These findings strengthen the hypothesis that miR-129 may serve as a molecular bridge between oncologic, vascular, and neurological pathologies, potentially reflecting shared inflammatory and metabolic mechanisms. Additional evidence supporting miR-129’s systemic role includes its inverse correlation with LDH and CRP levels, two well-established markers of systemic inflammation and tissue damage. This suggests that miR-129 may also function in regulating metabolic stress responses, reinforcing its relevance across multiple physiological systems. Our findings align with several previous reports. For example, Gao et al. (2016) [45] highlighted the tumor-suppressive function of miR-129 in various cancers, while Kaurani et al. [46], Loffreda et al. [47], and Qin et al. [48] collectively point to its role in neurodegeneration, vascular homeostasis, and cognitive regulation. This growing body of evidence supports the concept of miR-129 as a cross-system biomarker, with implications extending from oncology into vascular neurology.

This study has several notable strengths. It adopts a multidimensional approach, integrating molecular, oncologic, vascular, and neurologic data to explore the systemic relevance of miR-129. The use of gastric juice as a non-invasive biological matrix for miRNA analysis is particularly innovative and enhances clinical applicability. Additionally, the application of real-time quantitative PCR (RT-qPCR) ensures high sensitivity and specificity in miRNA quantification. The incorporation of established tumor markers (CEA and CA 19-9), imaging parameters (carotid index and MRI brain findings), and cognitive assessments (MMSE scores) allows for robust cross-validation of results across clinical domains. However, the study has important limitations. The small sample size (*n* = 38) limits statistical power and generalizability, while the single-center design may introduce institutional bias. Its cross-sectional nature precludes analysis of temporal changes or causal relationships in miR-129 expression. Moreover, functional validation experiments (e.g., luciferase assays or miR-129 overexpression/silencing) were not performed, limiting mechanistic interpretation. Potential confounding factors such as comorbidities (e.g., diabetes, cardiovascular disease) may also influence miRNA expression and were not fully controlled. Correlations with carotid index and neuroimaging are hypothesis-generating only; mechanistic links remain speculative pending longitudinal and functional validation

Despite these limitations, the study provides a strong foundation for future research on miR-129 as a multi-system biomarker.

## 5. Conclusions

Our study provides preliminary evidence suggesting that miR-129 expression is altered in patients with gastric adenocarcinoma compared to controls. Although the observed differences and the ROC analysis (AUC = 0.750) suggest potential biological relevance, the diagnostic utility remains limited at this stage. Importantly, our sample size was small and underpowered for definitive biomarker validation. While exploratory associations with neurovascular parameters were observed, these findings should be interpreted with caution, given the cross-sectional design and absence of functional validation. Overall, our findings should be viewed as hypothesis-generating. Further studies with larger, well-characterized cohorts and longitudinal follow-up are essential to confirm the diagnostic or prognostic role of miR-129 and to better understand its systemic and mechanistic implications in gastric cancer and beyond.

## Figures and Tables

**Table 2:** Mature MiR-129 sequence used in the study, suppressor of gastric cancer.

MiRNA Extracted from Gastric Juice	Role	Mature miRNA Sequence	Species	Level in Gastric Juice
Hsa-miR129-2-3p (miR129)	Anti-Oncogene	CUUUUUGCGGUCUGGGCUUGC	Human	Down-regulated in gastric cancer

**Table 3:** Student’s *t*-test for assessing differences between the mean carotid index and MIR-129.

*t*-Test: Two-Sample Assuming Equal Variances		
	MIR129	Carotid Index
Mean	8.87433592	0.57894737
Variance	35.0571548	0.25035562
Observations	38	38
Pooled Variance	17.6537552	
Hypothesized Mean Difference	0	
Df	74	
t Stat	8.605874	
P(T ≤ t) one-tailed	<0.05	
t Critical one-tailed	1.66570689	
P(T ≤ t) two-tailed	<0.05	
t Critical two-tailed	1.9925435	

**Table 4:** Student’s *t*-test for assessing differences between MRI and MIR-129.

*t*-Test: Two-Sample Assuming Equal Variances		
	mir_129	RMN (MRI)
Mean	8.87433592	0.84210526
Variance	35.0571548	0.13655761
Observations	38	38
Pooled Variance	17.5968562	
Hypothesized Mean Difference	0	
df	74	
t Stat	8.34632764	
P(T ≤ t) one-tailed	<0.05	
t Critical one-tailed	1.66570689	
P(T ≤ t) two-tailed	<0.05	
t Critical two-tailed	1.9925435	

## Data Availability

The datasets generated and analyzed during the current study are not publicly available due to institutional restrictions, but are available from the corresponding author upon reasonable request.

## References

[1] Sung H., Ferlay J., Siegel R.L., Laversanne M., Soerjomataram I., Jemal A., Bray F. (2021). Global cancer statistics 2020: GLOBOCAN estimates of incidence and mortality worldwide for 36 cancers in 185 countries. CA Cancer J. Clin..

[2] Azarbarzin S., Safaralizadeh R., Khojasteh M.B., Baghbanzadeh A., Baradaran B. (2020). Current perspectives on the dysregulated microRNAs in gastric cancer. Mol. Biol. Rep..

[3] Bautista-Sánchez D., Arriaga-Canon C., Pedroza-Torres A., De La Rosa-Velázquez I.A., González-Barrios R., Contreras-Espinosa L., Montiel-Manríquez R., Castro-Hernández C., Fragoso-Ontiveros V., Álvarez-Gómez R.M. (2020). The Promising Role of miR-21 as a Cancer Biomarker and Its Importance in RNA-Based Therapeutics. Mol. Ther. Nucleic Acids.

[4] Chen C., Tang X., Liu Y., Zhu J., Liu J. (2019). Induction/reversal of drug resistance in gastric cancer by non-coding RNAs (Review). Int. J. Oncol..

[5] Fang Y., Shen H., Li H., Cao Y., Qin R., Long L., Zhu X., Xie C., Xu W. (2013). MiR-106a confers cisplatin resistance by regulating PTEN/Akt pathway in gastric cancer cells. Acta Biochim. Biophys. Sin..

[6] Feng X., Zhu M., Liao B., Tian T., Li M., Wang Z., Chen G. (2020). Upregulation of miR-552 predicts unfavourable prognosis of gastric cancer and promotes the proliferation, migration and invasion of gastric cancer cells. Oncol. Res. Treat..

[7] Feng J., Guo J., Wang J.-P., Chai B.-F. (2020). MiR-129-5p inhibits proliferation of gastric cancer cells through targeted inhibition on HMGB1 expression. Eur. Rev. Med. Pharmacol. Sci..

[8] Ge X., Liu X., Lin F., Li P., Liu K., Geng R., Dai C., Lin Y., Tang W., Wu Z. (2016). MicroRNA-421 regulated by HIF-1α promotes metastasis, inhibits apoptosis, and induces cisplatin resistance by targeting E-cadherin and caspase-3 in gastric cancer. Oncotarget.

[9] Hao N.-B., He Y.-F., Li X.-Q., Wang K., Wang R.-L. (2017). The role of miRNA and lncRNA in gastric cancer. Oncotarget.

[10] Hou X., Zhang M., Qiao H. (2015). Diagnostic significance of miR-106a in gastric cancer. Int. J. Clin. Exp. Pathol..

[11] Jiang Z., Wang H., Li Y., Hou Z., Ma N., Chen W., Zong Z., Chen S. (2016). MiR-129-5p is down-regulated and involved in migration and invasion of gastric cancer cells by targeting interleukin-8. Neoplasma.

[12] Yu X., Luo L., Wu Y., Yu X., Liu Y., Yu X., Zhao X., Zhang X., Cui L., Ye G. (2013). Gastric juice miR-129 as a potential biomarker for screening gastric cancer. Med. Oncol..

[13] Huang Y.K., Yu J.C. (2015). Circulating microRNAs and long non-coding RNAs in gastric cancer diagnosis: An update and review. World J. Gastroenterol..

[14] Yuan H.L., Wang T., Zhang K.H. (2018). MicroRNAs as potential biomarkers for diagnosis, therapy and prognosis of gastric cancer. Onco Targets Ther..

[15] Liu Q., Jiang J., Fu Y., Liu T., Yu Y., Zhang X. (2018). MiR-129-5p functions as a tumor suppressor in gastric cancer progression through targeting ADAM9. Biomed. Pharmacother..

[16] Link A., Kupcinskas J. (2018). MicroRNAs as non-invasive diagnostic biomarkers for gastric cancer: Current insights and future perspectives. World J. Gastroenterol..

[17] Wang Q., Chen C., Ding Q., Zhao Y., Wang Z., Chen J., Jiang Z., Zhang Y., Xu G., Zhang J. (2020). METTL3-mediated m^6^A modification of HDGF mRNA promotes gastric cancer progression and has prognostic significance. Gut.

[18] Zhang M., Jiang D., Xie X., He Y., Lv M., Jiang X. (2019). MiR-129-3p inhibits NHEJ pathway by targeting SAE1 and represses gastric cancer progression. Int. J. Clin. Exp. Pathol..

[19] Roman Filip I., Morosanu V., Spinu D., Motoc C., Bajko Z., Sarmasan E., Roman C., Balasa R. (2024). Cervical Artery Dissections—A Demographical Analysis of Risk Factors, Clinical Characteristics Treatment Procedures, and Outcomes—A Single Centre Study of 54 Consecutive Cases. J. Pers. Med..

[20] Boicean A., Birsan S., Ichim C., Boeras I., Roman-Filip I., Blanca G., Bacila C., Fleaca R.S., Dura H., Roman-Filip C. (2023). Has-miR-129-5p’s Involvement in Different Disorders, from Digestive Cancer to Neurodegenerative Diseases. Biomedicines.

[21] Yang F., Sun Z., Wang D., Du T. (2022). MiR-106b-5p regulates esophageal squamous cell carcinoma progression by binding to HPGD. BMC Cancer.

[22] Roman-Filip I., Morosanu V., Bajko Z., Roman-Filip C., Balasa R.I. (2023). Non-Aneurysmal Perimesencephalic Subarachnoid Hemorrhage: A Literature Review. Diagnostics.

[23] Yang T.-S., Yang X.-H., Chen X., Wang X.-D., Hua J., Zhou D.-L., Zhou B., Song Z.-S. (2014). MicroRNA-106b in cancer-associated fibroblasts from gastric cancer promotes cell migration and invasion by targeting PTEN. FEBS Lett..

[24] Yu X., Song H., Xia T., Han S., Xiao B., Luo L., Xi Y., Guo J. (2013). Growth inhibitory effects of three miR-129 family members on gastric cancer. Gene.

[25] Fesler A., Zhai H., Ju J. (2014). miR-129 as a novel therapeutic target and biomarker in gastrointestinal cancer. Onco Targets Ther..

[26] Wang Q., Yu J. (2018). MiR-129-5p suppresses gastric cancer cell invasion and proliferation by inhibiting COL1A1. Biochem. Cell Biol..

[27] Yan L., Sun K., Liu Y., Liang J., Cai K., Gui J. (2017). MiR-129-5p influences the progression of gastric cancer cells through interacting with *SPOCK1*. Tumour Biol..

[28] Chen C., Jiang J., Fang M., Zhou L., Chen Y., Zhou J., Song Y., Kong G., Zhang B., Jiang B. (2020). MicroRNA-129-2-3p directly targets Wip1 to suppress the proliferation and invasion of intrahepatic cholangiocarcinoma. J. Cancer.

[29] Peng X., Wu X., Wu G., Peng C., Huang B., Huang M., Ding J., Mao C., Zhang H. (2023). MiR-129-2-3p Inhibits Esophageal Carcinoma Cell Proliferation, Migration, and Invasion via Targeting DNMT3B. Curr. Mol. Pharmacol..

[30] Xu H., Hu Y., Qiu W. (2017). Potential mechanisms of microRNA-129-5p in inhibiting cell processes including viability, proliferation, migration and invasiveness of glioblastoma cells U87 through targeting FNDC3B. Biomed. Pharmacother..

[31] Andronic M., Scripcariu D.V., Palaghia M.M., Trofin A.M., Bejan V., Scripcariu V. (2024). Clinical Pathological and Immunohistochemical Correlations in Gastric Cancer. Diagnostics.

[32] Liu H.-S., Xiao H.-S. (2014). MicroRNAs as potential biomarkers for gastric cancer. World J. Gastroenterol..

[33] Virgilio E., Giarnieri E., Giovagnoli M.R., Montagnini M., Proietti A., D’Urso R., Mercantini P., Balducci G., Cavallini M. (2018). Gastric Juice MicroRNAs as Potential Biomarkers for Screening Gastric Cancer: A Systematic Review. Anticancer. Res..

[34] Roman-Filip C., Catană M.G., Bereanu A., Lăzăroae A., Gligor F., Sava M. (2019). Therapeutic plasma exchange and double filtration plasmapheresis in severe neuroimmune disorders. Acta Clin. Croat..

[35] Fujimoto S., Kitsukawa U., Itoh K. (1979). Carcinoembryonic antigen (CEA) in gastric juice or feces as an aid in the diagnosis of gastrointestinal cancer. Ann. Surg..

[36] Wada N., Kurokawa Y., Miyazaki Y., Makino T., Takahashi T., Yamasaki M., Nakajima K., Takiguchi S., Mori M., Doki Y. (2017). The characteristics of the serum carcinoembryonic antigen and carbohydrate antigen 19-9 levels in gastric cancer cases. Surg. Today.

[37] Xu W., Yang Z., Lu N. (2016). Molecular targeted therapy for the treatment of gastric cancer. J. Exp. Clin. Cancer Res..

[38] Kang H.S., Jang S.G., Kwon S.Y., Park Y.S., Green J.E., Kim H.K., Ro J. (2014). MicroRNA signature for HER2-positive breast and gastric cancer. Anticancer. Res..

[39] Xiao B., Guo J., Miao Y., Jiang Z., Huan R., Zhang Y., Li D., Zhong J. (2009). Detection of miR-106a in gastric carcinoma and its clinical significance. Clin. Chim. Acta..

[40] Wei L., Sun J., Zhang N., Zheng Y., Wang X., Lv L., Liu J., Xu Y., Shen Y., Yang M. (2020). Noncoding RNAs in gastric cancer: Implications for drug resistance. Mol. Cancer.

[41] Xie Y., Shi L., He X., Luo Y. (2021). Gastrointestinal cancers in China, the USA, and Europe. Gastroenterol. Rep..

[42] Han C., Xu T., Zhang Q., Liu J., Ding Z., Hou X. (2021). The New American Joint Committee on Cancer T staging system for stomach: Increased complexity without clear improvement in predictive accuracy for endoscopic ultrasound. BMC Gastroenterol..

[43] Yu J., Zhang X., Ma Y., Li Z., Tao R., Chen W., Xiong S., Han X. (2021). *MiR-129-5p* Restrains Apatinib Resistance in Human Gastric Cancer Cells Via Downregulating *HOXC10*. Cancer Biother. Radiopharm..

[44] R Development Core Team (2011). R: A Language and Environment for Statistical Computing.

[45] Gao Y., Feng B., Han S., Lu L., Chen Y., Chu X., Wang R., Chen L. (2016). MicroRNA-129 in Human Cancers: From Tumorigenesis to Clinical Treatment. Cell Physiol. Biochem..

[46] Kaurani L., Pradhan R., Schröder S., Burkhardt S., Schuetz A.-L., Krüger D.M., Pena T., Heutink P., Sananbenesi F., Fischer A. (2025). A role for astrocytic miR-129-5p in frontotemporal dementia. Transl. Psychiatry.

[47] Loffreda A., Nizzardo M., Arosio A., Ruepp M.-D., Calogero R.A., Volinia S., Galasso M., Bendotti C., Ferrarese C., Lunetta C. (2020). miR-129-5p: A key factor and therapeutic target in amyotrophic lateral sclerosis. Prog. Neurobiol..

[48] Qin Q., Zhang H., Li X., Ruan H., Liu S., Chen Y., Xu Z., Wang Y., Yan X., Jiang X. (2025). MiR-129-5p alleviates depression and anxiety by increasing astrocyte ATP production partly through targeting deubiquitinase Mysm1. PLoS ONE.

[49] Wu X., Tang H., Liu P., Zheng L. (2014). MiR-21 expression in gastric carcinoma and its correlation with clinicopathological parameters and prognosis. Int. J. Clin. Exp. Pathol..

[50] Zhang Y., Guo J., Li D., Xiao B., Miao Y., Jiang Z. (2012). Down-regulation of miR-421 contributes to the proliferation and invasion of gastric cancer cells. Clin. Investig. Med..

